# Inhibition of astroglial hemichannels prevents synaptic transmission decline during spreading depression

**DOI:** 10.1186/s40659-024-00519-9

**Published:** 2024-06-12

**Authors:** Juan E. Tichauer, Matías Lira, Waldo Cerpa, Juan A. Orellana, Juan C. Sáez, Maximiliano Rovegno

**Affiliations:** 1 Departamento de Medicina Intensiva, Facultad de Medicina, Pontificia Universidad Católica de Chile, Santiago, Chile; 2 Departamento de Biología Celular y Molecular, Facultad de Ciencias Biológicas, Pontificia Universidad Católica de Chile, Santiago, Chile; 3 Facultad de Ciencias Biológicas, Pontificia Universidad Católica de Chile, Santiago, Chile; 4 Departamento de Neurología, Escuela de Medicina, Facultad de Medicina, Pontificia Universidad Católica de Chile, Santiago, Chile; 5 Centro Interdisciplinario de Neurociencias, Pontificia Universidad Católica de Chile, Santiago, Chile; 6 Departamento de Fisiología, Facultad de Ciencias Biológicas, Pontificia Universidad Católica de Chile, Santiago, Chile; 7 Instituto de Neurociencias, Centro Interdisciplinario de Neurociencias de Valparaíso, Universidad de Valparaíso, Valparaíso, Chile

**Keywords:** Spreading depression, Connexin-43, Pannexin-1, Astrocyte, Neurons, Synaptic transmission

## Abstract

**Background:**

Spreading depression (SD) is an intriguing phenomenon characterized by massive slow brain depolarizations that affect neurons and glial cells. This phenomenon is repetitive and produces a metabolic overload that increases secondary damage. However, the mechanisms associated with the initiation and propagation of SD are unknown. Multiple lines of evidence indicate that persistent and uncontrolled opening of hemichannels could participate in the pathogenesis and progression of several neurological disorders including acute brain injuries. Here, we explored the contribution of astroglial hemichannels composed of connexin-43 (Cx43) or pannexin-1 (Panx1) to SD evoked by high-K^+^ stimulation in brain slices.

**Results:**

Focal high-K^+^ stimulation rapidly evoked a wave of SD linked to increased activity of the Cx43 and Panx1 hemichannels in the brain cortex, as measured by light transmittance and dye uptake analysis, respectively. The activation of these channels occurs mainly in astrocytes but also in neurons. More importantly, the inhibition of both the Cx43 and Panx1 hemichannels completely prevented high K^+^-induced SD in the brain cortex. Electrophysiological recordings also revealed that Cx43 and Panx1 hemichannels critically contribute to the SD-induced decrease in synaptic transmission in the brain cortex and hippocampus.

**Conclusions:**

Targeting Cx43 and Panx1 hemichannels could serve as a new therapeutic strategy to prevent the initiation and propagation of SD in several acute brain injuries.

**Supplementary Information:**

The online version contains supplementary material available at 10.1186/s40659-024-00519-9.

## Introduction

Spreading depression (SD) encompasses waves of partial or complete gray matter depolarization characterized by a focal negative surface potential shift that slowly propagates through contiguous tissue at a rate of 2–5 mm/min [1–3]. At the cellular level, SDs cause an almost complete breakdown of electrochemical gradients across the cell membrane [4], suppression of electrical activity, cellular swelling, and changes in regional blood flow [5]. Extensive research has been conducted to understand the ionic shifts that underlie SD, identify the molecular mechanisms responsible for its propagation, and explore the implications of these events in various acute brain injuries such as subarachnoid and intracerebral hemorrhage, ischemic stroke, traumatic brain injury (TBI) and brain death.

While early studies primarily focused on neuronal activity in understanding SD mechanisms, recent attention has shifted towards the role of astrocytes in this phenomenon, prompting ongoing investigation and scrutiny [6]. Astrocytes play diverse and critical roles in the central nervous system (CNS), such as modulating synaptic transmission, locally regulating blood flow, and preserving extracellular fluid homeostasis in the brain [7–9]. Although it was initially postulated that depolarization of astrocyte networks plays a significant role in the extracellular voltage shifts recorded during SD [3, 10], further studies revealed that astrocytes follow rather than lead to SD [10, 11]. Nevertheless, it is essential to acknowledge that astrocyte function could still play a pivotal role in determining the severity or outcome of SD [6]. Indeed, SD induces significant K^+^ uptake into astrocytes, leading to astrocyte swelling [12], whereas pronounced Ca^2+^ waves are propagated through astrocyte networks with SDs [13–15]. Intercellular communication mediated by connexin-based channels is critical not only for the propagation of Ca^2+^ waves among astrocytes but also for the spatial buffering of K^+^ in the brain [9, 16–18]. Connexins are transmembrane proteins that create two pathways for intercellular communication: (1) gap junctional channels (GJCs), which are formed by the docking of two connexons or hemichannels positioned at opposite membranes between adjacent cells, and (2) hemichannels, which are situated at unopposed regions of cell surfaces [19]. Each hemichannel is an array of six connexins surrounding a central pore. GJCs enable direct cell-to-cell exchange of small molecules, ions, and second messengers, such as Ca^2+^ and inositol trisphosphate (IP_3_) [20]. Hemichannels permit the exchange of molecules and ions between the cytoplasm and the external medium, supporting autocrine and paracrine actions [21]. On the other hand, pannexins, another family of transmembrane proteins with three members (Panx 1–3) [22], form GJCs [23] and hemichannels (also known as pannexons) [24]. Pannexins share a similar topology to connexins but have significant divergence in amino acid sequence [25]. Most astrocytes express GJCs formed by connexin-43 (Cx43) and Cx30, whereas Cx43 and Panx1 have been shown to form functional hemichannels in these glial cells [26].

Cellular signaling, facilitated by the opening of astroglial hemichannels, underlies critical biological processes within the nervous system [27, 28]. The latter encompasses neuronal oscillations [29], the glutamate–glutamine shuttle [30], memory [31–34], synaptic transmission and plasticity [31, 35, 36]. Despite these vital functions, the uncontrolled activation of astroglial hemichannels leads to osmotic and ionic imbalance, cytoplasmic Ca^2+^ overload, and the release of large quantities of potentially toxic molecules, such as glutamate, ATP, and D-serine [26, 37].

The involvement of connexins and pannexins in SD, as well as in acute brain injuries, continues to be a topic of debate, with conflicting results regarding their impact on SD generation. These controversial findings could be attributed to the fact that the approaches employed (pharmacological inhibitors and knockout strategies) target both hemichannels and GJCs composed of Cx43 [38] [39]. On the other hand, blocking Panx1 hemichannel activity using probenecid and the specific extracellular peptide ^10^panx1 did not prevent SD in mice but effectively hindered Panx1 hemichannel activation associated with SD [40]. Therefore, the contribution of astroglial hemichannels to the pathogenesis and progression of SD is still poorly understood. This study aimed to determine the contribution of astroglial hemichannels in SD using an ex vivo model of this phenomenon. Here, we reported that SD evoked by brief focal high [K^+^] stimulation rapidly increases the activity of Cx43 and Panx1 hemichannels in the brain cortex. More importantly, the inhibition of these hemichannels completely prevented high [K^+^]-induced SD and the reduction of synaptic transmission evoked by SD.

## Materials and methods

### Reagents and antibodies

The mimetic peptides TAT-Gap19 (YGRKKRRQRRR-KQIEIKKFK, the intracellular loop domain of Cx43) and ^10^panx1 (WRQAAFVDSY, the first extracellular loop domain of Panx1) were purchased from SBS Genetech Co., Ltd. (SBS Genetech, Beijing, China) with a purity > 95%. Ethidium (Etd) bromide, L-2 aminoadiptic acid (L-AA), tetrodotoxin (TTX), MK801, lanthanum chloride (La^3+^), probenecid (PBC) and Fluoromount were purchased from Sigma‒Aldrich (Sigma‒Aldrich, St. Louis, MO, USA). Picrotoxin was obtained from Tocris (PTX, Tocris, Bristol, UK). Normal goat serum, Hoechst 33342, rat anti-glial fibrillary acidic protein (GFAP) monoclonal antibody, rabbit anti-NeuN oligoclonal antibody, goat anti-rat Alexa Fluor 488, and goat anti-rabbit Alexa 647 were obtained from Thermo Fisher (Thermo Fisher, Waltham, MA, USA).

### Mice

Animal experimentation was conducted following the guidelines for the care and use of experimental animals of the US National Institutes of Health (NIH), the ad hoc committee of the Chilean government (ANID), the Bioethics and Care of Laboratory Animals Committee of the Pontificia Universidad Católica de Chile (PUC) (protocol #170518005) and the European Community Council Directives of November 24th, 1986. Male C57BL/6J mice, 8–12 weeks old from the PUC animal care unit (CIBEM), were housed in cages in a temperature-controlled (24 °C) and humidity-controlled vivarium under a 12 h light/dark cycle (lights on 8:00 AM) with ad libitum access to food and water.

### Acute brain slices

The animals were deeply anesthetized with isoflurane and decapitated. The brains were removed from the skull and placed in ice-cold oxygenated artificial cerebrospinal fluid (aCSF) containing (in mM) NaCl (125), KCl (2.5), glucose (25), NaHCO_3_ (25), NaH_2_PO_4_ (1.25), CaCl_2_ (2), and MgCl_2_ (1) bubbled with 95% O_2_/5% CO_2_, pH 7.4. The rostral and caudal parts of the brains were removed with a razor blade, and the brains were gently glued to a holder in a tray filled with slicing buffer (in mM): 85 NaCl, 3 KCl, 0.5 CaCl_2_, 3.5 MgSO_4_, 1.25 NaH_2_PO_4_, 25 NaHCO_3_, 10 glucose, 222 sucrose, 0.5 Na-ascorbate, and 3 Na-pyruvate, saturated with 95% O_2_ and 5% CO_2_ at room temperature. Coronal slices (300 µm, bregma − 1.8 mm to − 3.3 mm, mainly the somatosensorial and visual cortex) were sectioned using a 5100mz Campden Instruments vibratome (Loughborough, UK). Once the slices were freed, they were separated into two hemispheres (hemi-brain slices), one for the experimental treatment group and the other for the control group, using a scalpel. Then, the slices were transferred to separate chambers for stabilization with aCSF saturated with 95% O_2_ and 5% CO_2_ for at least 1 h before the experiments.

### Experimental treatments

Some brain slices were treated with the following blockers in aCSF before experiments: 1 mM L-AA for 1 h, 1 µM TTX for 15 min, 200 µM La^3+^, 500 µM PBC for 30 min, 300 µM TAT-Gap19 for 30 min, 200 µM ^10^panx1 for 30 min or 400 µM MK801 for 30 min.

### Induction of spreading depression

**Fig. 1:**
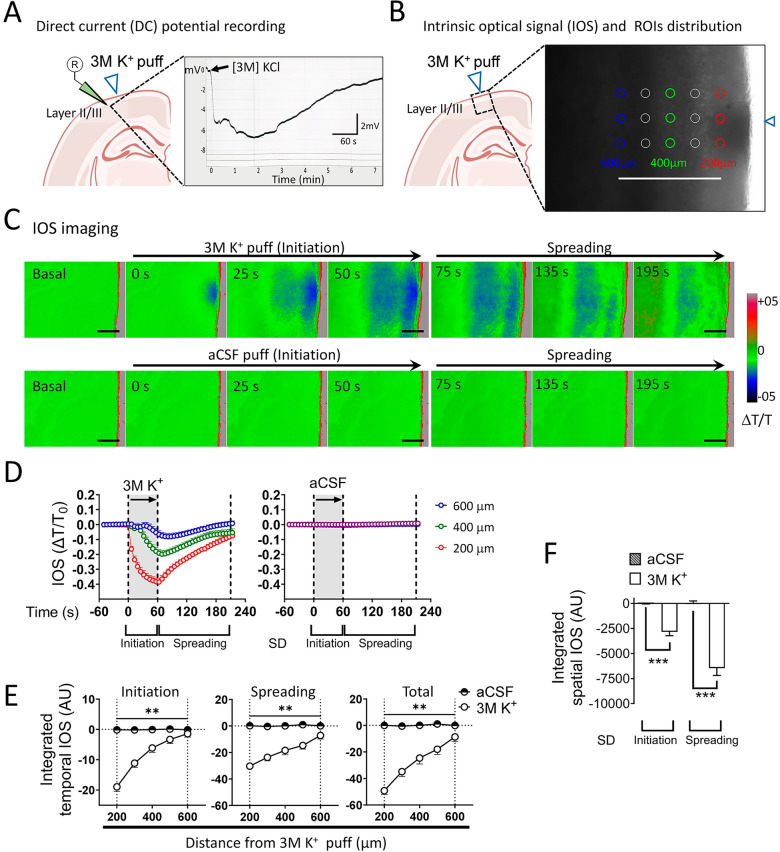
Characterization of the spreading depression evoked by a brief focal high [K^+^] puff in acute brain slices. **A** A schematic showing the site of focal high [K^+^] puff as well as the position of the DC recording electrode on cortical layers II/III. The inset shows a representative DC trace of the negative shift in the extracellular potential induced by the focal high [K^+^] puff. **B** A schematic showing the site of focal high [K^+^] puff and the area where the circular ROIs were placed in cortical layers II/III. The inset shows the site of focal high [K^+^] puff (blue triangle) and the distribution of circular ROIs (35 µm diameter) in a grid position for IOS analysis. **C** Top panel. Representative time-lapse recordings of the IOS showing the initiation and spreading of a single focally induced SD by a 3 M [K^+^] puff. Bottom panel. Time-lapse recording of the IOS showing the absence of SD when the puff is performed with normal [K^+^]-aCSF. Scale bar, 100 µm. **D** Representative plots of relative changes in the IOS over time induced by 3 M [K^+^] (left) or normal [K^+^]-aCSF (right) puffs at different distances from the site of stimulation_._ The gray areas between the dashed lines delimit the initiation and spreading phases of the IOS wave. **E** Averaged data of the integrated temporal IOS (from plots shown in **D**) induced by 3 M [K^+^] (white circles) or normal [K^+^]-aCSF (half black/white circles) puffs during the initiation, spreading or both phases of the SD wave at different distances from the site of stimulation. **P < 0.01 for the effect of 3 M [K^+^] puff compared to aCSF puff during the different phases of the IOS wave (Mann–Whitney nonparametric test). **F** Averaged data of integrated spatial IOSs (from plots shown in **E** induced by 3 M [K^+^] or normal [K^+^]-aCSF puffs during the initiation and spreading phases of SD waves at different distances from the site of stimulation. ***P < 0.001 for the effect of 3 M [K^+^] puff compared to aCSF puff during the different phases of the IOS wave (Mann–Whitney nonparametric test). The values are expressed in arbitrary units (A.U.). 3 M [K^+^], n = 22 mice; aCSF, n = 6 mice

A brief puff of high [K^+^] was used for the focal induction of SD [41, 42]. Briefly, brain slices were placed in a 30 mm Petri dish filled with 3 ml aCSF and fixed under a standard harp slice grid (ALA Scientific Instruments, USA). A glass borosilicate micropipette (0.5–1 MΩ resistance, tip size 10–20 mm) filled with 3 M KCl or aCSF was placed 100 µm from the brain cortex border in the slices. KCl was applied to the slices with a pulse (20 PSI for 60 s) using a Picospritzer II (Parker, NH, USA) that delivered a total volume of ~ 30 nL (Fig. 1). Unless otherwise stated, SD induction by focal high [K^+^] puff without any blocker was considered the control condition.

### Intrinsic optical signal recordings

Acute slices were trans-illuminated by a white-light source (Zeiss SNT tungsten, 12 V 100 W), and the intrinsic optical signal (IOS) was collected on a Zeiss Axio Observer D.1. An inverted microscope with a 10x/0.25 A-plan Zeiss objective lens and an AxioCam MRm monochrome digital camera R3.0 (Carl Zeiss AG, Zeiss, Oberkochen, Germany) was used. Grayscale values were quantified in 24 circular regions of interest (ROIs) (35 µm diameter) drawn at multiple distances from the K^+^ puff stimulation site in a 300 µm × 600 µm grid (Fig. 1B). To examine alterations in the IOS after SD induction, light transmittance images were recorded at 6 s intervals for 5.5 min (Fig. 1C). Offline image processing was performed with ZEN Pro software (Zen 2.3 [blue edition], Carl Zeiss AG, Oberkochen, Germany). The first 10 frames of each image series were averaged and used as the baseline for subsequent baseline subtraction and normalization. The IOS is expressed as the ratio of the change in LT with respect to baseline (T_0_). That is, IOS = (T-T_0_)/T_0_ = ΔT/T_0_ (Fig. 1D). To analyze changes in the IOS, first, the net area under the curve was determined for ROIs at different distances from the focal high [K^+^] puff site over time (integrated temporal IOS; Fig. 1E). Second, the net area under the curve from the integrated temporal IOS (integrated spatial IOS; Fig. 1F) was determined during the focal high [K^+^] puff (initiation phase) and the posterior 2.5 min when 95% of the optic signal reached the basal level (spreading phase) [43].

### Dye uptake recordings

Acute slices were incubated with 15 μM Etd in aCSF for 3 min before high [K^+^]_e_ focal stimulation. Then, the slices were mounted on the stage of a Zeiss Axio Observer D.1. An inverted microscope with a 10x/0.25 A-plan Zeiss objective lens and an AxioCam MRm monochrome digital camera R3.0 (Carl Zeiss AG, Zeiss, Oberkochen, Germany) was used. Images were captured every 6 s for 5.5 min (exposure time = 0.5 s; excitation and emission wavelengths were 528 nm and 598 nm, respectively). Offline image processing was performed with ZEN Pro software (Zen 2.3 [blue edition], Carl Zeiss AG, Oberkochen, Germany). Grayscale values were quantified in at least 35 nuclear ROIs (15 µm diameter) distributed across cortical layers between 200 µm and 500 µm from the focal high [K^+^] puff stimulation site. The increase in Etd fluorescence was normalized and is expressed as a percentage of the maximal fluorescence reached by the control condition produced by focal high [K^+^] puffs, as shown in the time-lapse graphs (Fig. 4C). The area under the curve (AUC) was determined from time-lapse images (integrated Etd uptake; Fig. 4D) to analyze Etd uptake. Moreover, alterations in the slope of the Etd uptake curve were determined through linear regression analysis of the data obtained during the initiation or spreading phases after the focal high [K^+^] puff (Fig. 4E**)**. All microscope images were analyzed using Zeiss software Zen Blue Edition (Carl Zeiss Microscopy, Oberkochen, Germany).

### Immunohistochemistry

In some experiments on Etd uptake, the slices were subjected to immunohistochemistry to allow cellular identification. In brief, the slices were washed three times with aCSF and fixed at 4 °C with 4% sucrose in 4% paraformaldehyde overnight. The slices were rinsed once for 5 min with 0.1 mM glycine in PBS and then twice with PBS for 10 min with gentle agitation. Then, the slices were incubated for 30 min each with a blocking solution (PBS containing 5% NGS and 0.1% Triton-X 100) at room temperature and then incubated overnight at 4 °C with a cell-specific antibody to identify astrocytes (1:500 rat anti-GFAP monoclonal antibody) and neurons (1:250 rabbit anti-NeuN oligoclonal antibody) diluted in blocking buffer. Then, the slices were washed 3 times for 10 min with PBS and incubated for 2 h at room temperature with goat anti-rat Alexa Fluor 488 (1:500) and goat anti-rabbit Alexa Fluor 647 antibodies. After 3 washes (10 min each), the slices were mounted in Fluoromount, cover-slipped, and examined under a confocal microscope (Airyscan Zeiss). Images were taken with a 20 × objective and analyzed with Fiji software (National Institute of Health, Bethesda, MD, USA). The number of cells stained for Etd/GFAP or Etd/NeuN in cortical layer I or cortical layer II/II was recorded for at least six fields (400 × 400 µm) per condition. The median number of cells per field was compared for each condition.

### Electrophysiological recordings

To obtain the local field potential, we used borosilicate micropipettes with resistances of approximately 5 MΩ made with a P-97 puller (Sutter, Instrument, Novato, CA, USA). The micropipettes were backfilled with aCSF, and an Ag/AgCl metal electrode contacted the aCSF inside the micropipette. Extracellular DC potential was acquired using a 16-bit data acquisition system (Digidata 1322A; Axon Instruments) and amplifier (Multiclamp 700B; Molecular Devices, San Jose, CA, USA). The data were recorded and analyzed offline with pClamp 10 software (Molecular Devices, San Jose, CA, USA). In these experiments, 10 μM PTX was added to suppress inhibitory GABA(A) transmission. Slices were transferred to an experimental chamber (2 ml), superfused (3 ml/min, at room temperature) with aCSF, saturated with 95% O_2_ and 5% CO_2_, and visualized by transillumination with a binocular microscope (Amscope, Irvine, CA, USA). To evoke field excitatory postsynaptic potentials (fEPSPs), we stimulated the cells with concentric bipolar electrodes (tungsten, 125 µm OD, microprobes) connected to an isolation unit (Isoflex, AMPI, Jerusalem, Israel). Layers II/III and V of the brain cortex or the Shaffer collaterals of the hippocampus were stimulated. The recording electrode was within 100–200 µm of the stimulation site [44]. The records were filtered at 2.0–3.0 kHz, recorded at 4.0 kHz using an A/D converter (National Instrument, Austin, TX, USA), and stored with the WinLTP program [45]. Baseline excitatory synaptic transmission was measured using an input/output curve protocol consisting of 10 stimuli ranging from 200 to 900 μA (the interval between stimuli was 10 s). The data were analyzed offline with pClamp 10 software (Molecular Devices, San Jose, CA, USA).

### Statistical analysis

The data are expressed as the mean $\pm$ SEM or median (IQR); *n* refers to the number of independent experiments performed. The error bars in the graphs represent the means ± SEMs. The normality of the data distribution was assessed by the Shapiro‒Wilk normality test. The results were analyzed using a Mann–Whitney nonparametric test to compare two means or one-way ANOVA for three or more groups with Dunnett's post hoc test for multiple comparisons, according to their normal distribution. GraphPad Prism v.10.1.0 (La Jolla, CA, USA) was used for statistical analysis and graphing. P values < 0.05 were considered to indicate statistical significance.

## Results

### Astroglial hemichannels contribute to the spreading depression evoked by high [K^+^] stimulation in the brain cortex

In normally metabolizing tissue, SD is usually triggered by depolarizing stimuli that elevate [K^+^]_e_ beyond a critical threshold [5]. To clarify the uncertainty regarding the involvement of astroglial hemichannels in this phenomenon, we employed a widely used ex vivo technique to induce SD: a brief focal high [K^+^] puff [41, 42]. SD is characterized by transmembrane ion flows, transient membrane potential depolarization, cell swelling, and shrinkage of the extracellular space [46]. These changes were measured by recording the local extracellular field potential and light transmittance (IOS) in mouse brain slices containing the somatosensory and visual cortex (Fig. 1A–C). A recording electrode on layer II/III monitored the extracellular field potential evoked by a micropipette containing 3 M K^+^ (focal high [K^+^] puff) positioned 100 µm from the brain cortex (Fig. 1A). As expected, a sudden negative shift of -6.76 ± 1.5 mV correlated temporally and spatially with the propagation of a wave of decreased IOS away from the induction site (Fig. 1B, C). The high [K^+^]-induced negative shift in the extracellular field potential progressively returned to baseline within 3–7 min following the focal high [K^+^] puff (Fig. 1A). IOS imaging revealed that the decrease in light transmittance peaked during high [K^+^] stimulation (initiation phase) (Fig. 1D). After that, within 2.5 min, 95% of the IOSs returned to baseline (spreading phase) (Fig. 1D). Indeed, temporal integration of the IOS during the initiation and spreading phases showed that the decrease in light transmittance was inversely proportional to the distance from the stimulation site (Fig. 1E). These kinetics aligned with classical changes in the IOS linked to SD induced in normal extracellular medium using submerged brain slices [10, 43, 47, 48]. Importantly, temporal and spatial integration of the IOS demonstrated that a high [K^+^]-induced decrease in light transmittance did not occur when the slices were stimulated only with a physiological aCSF puff, confirming the high [K^+^] dependency of this ex vivo SD model (Fig. 1D–F).

**Fig. 2:**
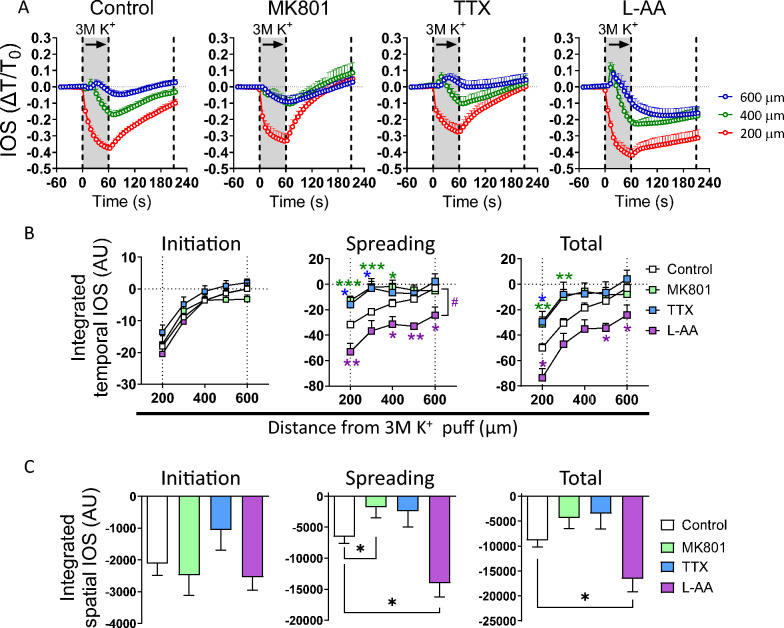
Neurons and astrocytes contribute in opposite ways to the spreading depression caused by high [K^+^] in the brain cortex. **A** Representative plots of relative changes in the IOS over time induced by 3 M [K^+^] puff at different distances from the site of stimulation in brain slices under control conditions or treated with MK801 (400 µM), TTX (1 µM), or L-AA (1 mM). The gray areas between the dashed lines delimit the initiation and spreading phases of the IOS wave. **B** Averaged data of the integrated temporal IOS (from plots in **A**) induced by 3 M [K^+^] puff during the initiation, spreading, or both phases of the SD wave at different distances from the site of stimulation in brain slices under control conditions or treated with MK801 (400 µM), TTX (1 µM), or L-AA (1 mM). *P < 0.05, **P < 0.01, ***P < 0.001, for the effect of neuron or astrocyte inhibition compared to the respective control condition in each ROI during the different phases of the IOS wave (Mann–Whitney nonparametric test); #*P* < 0.05 for the effect along ROIs (Mann–Whitney nonparametric test). **C** Averaged data of the integrated spatial IOS (from plots in **B**) induced by 3 M [K^+^] puff during the initiation, spreading, or both phases of the SD wave at different distances from the site of stimulation in brain slices under control conditions or treated with MK801 (400 µM), TTX (1 µM), or L-AA (1 mM). *P < 0.05 for the effect of neuron or astrocyte inhibition compared to the respective control condition during the different phases of the IOS wave (Mann–Whitney nonparametric test). The values are expressed in arbitrary units (A.U.). n = 7 mice per group

To evaluate the global participation of neurons in high [K^+^]-induced SD, both MK801 (a nonspecific NMDA receptor blocker), a known SD inhibitor [49], and TTX, a specific voltage-gated Na^+^ channel blocker [50], were used. MK801 (400 µM) or TTX (1 µM) significantly prevented the high [K^+^]-induced decrease in the IOS during spreading but not during the initiation phase (Fig. 2A, B). Next, we asked whether the inhibition of astrocyte-related functions could impact the SD elicited by focal high [K^+^] puff. For that purpose, we used L-AA, a gliotoxin selectively incorporated into astrocytes by the cystine–glutamate antiporter. It causes astrocyte toxicity by disturbing glutamate-dependent metabolism and protein synthesis, leading to loss of cellular integrity [51, 52]. L-AA has been previously used to selectively block astrocyte-mediated functions in the adult rodent brain [53–55]. In contrast to MK801 or TTX, L-AA exacerbated the high [K^+^]-induced decrease in IOS during the spreading phase of light transmittance records (Fig. 2A–C). Interestingly, these data indicate that neuronal inhibition reduces the SD evoked by high [K^+^] stimulation, whereas ablation of astrocytic function has the opposite effect.

**Fig. 3:**
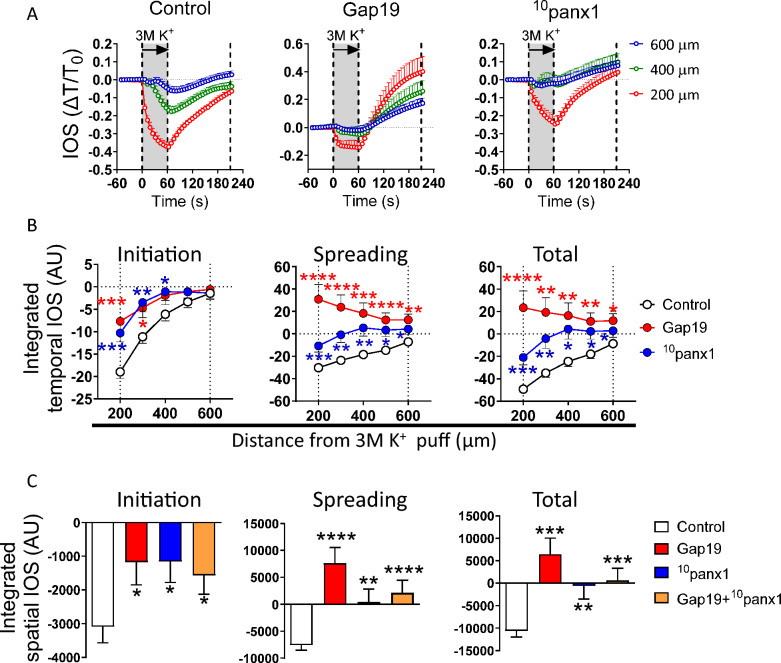
Connexin-43 and pannexin-1 hemichannels participate in the spreading depression evoked by high [K^+^] in the brain cortex. **A** Representative plots of relative changes in the IOS over time induced by 3 M [K^+^] puff at different distances from the site of stimulation in brain slices under control conditions or treated with Gap19 (300 µM), ^10^panx1 (200 µM) or both. The gray areas between the dashed lines delimit the initiation and spreading phases of the IOS wave. **B** Averaged data of the integrated temporal IOS (from plots in **A**) induced by 3 M [K^+^] puff during the initiation, spreading or both phases of the SD wave at different distances from the site of stimulation in brain slices under control conditions or treated with Gap19 (300 µM), ^10^panx1 (200 µM) or both. *P < 0.05, **P < 0.01, ***P < 0.001, ****P < 0.0001 for the effect of Cx43 or Panx1 hemichannel inhibition compared to the respective control conditions in each ROI during the different phases of the IOS wave (Mann–Whitney nonparametric test). **C** Averaged data of the integrated spatial IOS (from plots in **B** induced by 3 M [K^+^] puff during the initiation, spreading, or both phases of the SD wave at different distances from the site of stimulation in brain slices under control conditions or treated with Gap19 (300 µM), ^10^panx1 (200 µM) or both. *P < 0.05, **P < 0.01, ***P < 0.001, ****P < 0.0001 for the effect of Cx43 or Panx1 hemichannel inhibition compared to the respective control conditions during the different phases of the IOS wave (Mann–Whitney nonparametric test). The values are expressed in arbitrary units (A.U.). Control, n = 22; Gap19, n = 15; ^10^panx1, n = 15; Gap19 + ^10^panx1 = 10, mice per group

Previous evidence has shown that a high [K^+^]_e_ augments the activity of Cx43 and Panx1 hemichannels in astrocytes [56, 57]. Considering the enhancing effect of astrocytic inhibition on high [K^+^]-induced SD, we further examined whether Cx43 or Panx1 hemichannels could also influence SD in our system. Accordingly, we used TAT-Gap19 (Gap19) and ^10^panx1, two specific mimetic peptide blockers that bind the intracellular and first extracellular loops of Cx43 and Panx1, respectively [58, 59]. Notably, ^10^panx1 (200 µM) completely prevented the high [K^+^]-induced decrease in IOS during both the initiation and spreading phases of light transmittance records (Fig. 3A–C). Similar effects were detected with 500 µM PBC (Supplementary Fig. 1), a well-known inhibitor of Panx1 hemichannels [60]. Moreover, while Gap19 (300 µM) significantly decreased the IOS in the initiation phase, it led to an increasing trend in the IOS during the spreading phase, particularly closer to the high [K^+^] stimulation site (Fig. 3A–C). In addition, 200 µM La^3+^, a general blocker of connexin hemichannels and Ca^2+^-permeable channels [61, 62], decreased the IOS in the initiation and spreading phases (Supplementary Fig. 1). Interestingly, the combination of Gap19 and ^10^panx1 had a preventive effect similar to that observed with ^10^panx1 alone (Fig. 3C). The latter indicates that ^10^panx1 neutralized the Gap19-mediated increase in IOS during the spreading phase. Taken together, these results suggest that the Cx43 and Panx1 hemichannels contribute to the SD evoked by focal high [K^+^] stimulation in the brain cortex.

### High [K^+^]-induced spreading depression increases astroglial and neuronal hemichannel activity in the brain cortex

**Fig. 4:**
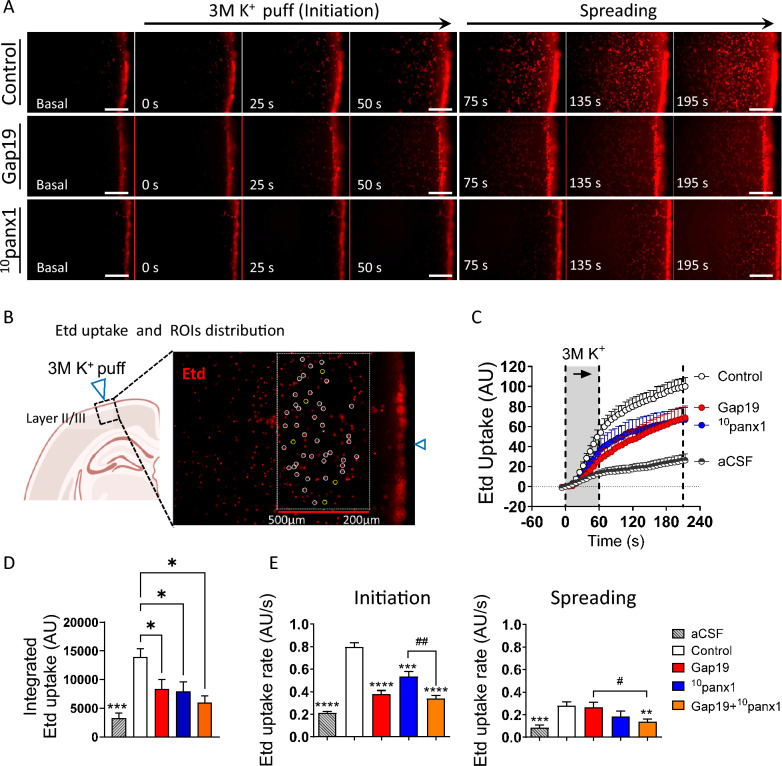
High [K^+^]-induced spreading depression activates connexin-43 and pannexin-1 hemichannels in the brain cortex. **A** Representative time-lapse images of Etd uptake in the initiation and spreading phases of SD induced by 3 M [K^+^] puff in brain slices under control conditions (top panel) or after treatment with Gap19 (300 µM) (middle panel) or ^10^panx1 (200 µM) (bottom panel). Scale bar, 100 µm. **B** Schematic showing the site of focal high [K^+^] puff as well and the area where the circular ROIs were placed in cortical layers II/III. The inset shows the site of focal high [K^+^] puff (blue triangle) and an example of the distribution of circular ROIs (15 µm diameter) on cell nuclei for Etd uptake analysis. **C** Averaged plots of Etd uptake normalized to that in control brain slices (white circles) induced by 3 M [K^+^] puff in brain slices treated with Gap19 (300 µM) (red circles) or ^10^panx1 (200 µM) (blue circles). In addition, the effect of normal [K^+^]-aCSF is shown (half black/white circles). The gray areas between the dashed lines delimit the initiation and spreading phases of the Etd uptake wave. **D** Averaged data of integrated Etd uptake induced by 3 M [K^+^] puff in brain slices under control conditions or after treatment with Gap19 (300 µM), ^10^panx1 (200 µM) or both. In addition, the effect of normal [K^+^]-aCSF is shown. *P < 0.05, ***P < 0.001, for the effect of Cx43 or Panx1 hemichannel inhibition compared to the respective control conditions during the different phases of SD (one-way ANOVA followed by Dunnet's post hoc test). **E** Average Etd uptake rate induced by 3 M [K^+^] puff during the initiation and spreading phases in brain slices under control conditions or after treatment with Gap19 (300 µM), ^10^panx1 (200 µM) or both. *P < 0.05, **P < 0.01, ***P < 0.001, ****P < 0.0001 for the effect of Cx43 or Panx1 hemichannel inhibition compared to the respective control conditions during the different phases of SD (one-way ANOVA followed by Dunnet's post hoc test); ^#^*P* < 0.05, ^##^*P* < 0.01 for the effect of Cx43 or Panx1 hemichannel inhibition compared to the effect of Gap19 + ^10^panx1 peptides (one-way ANOVA followed by Dunett post hoc test). In addition, the effect of normal [K^+^]-aCSF is shown. aCSF, n = 4; Control, n = 22; Gap19, n = 15; ^10^panx1, n = 15; Gap19 + ^10^panx1, n = 10 mice per group

To evaluate whether our ex vivo model of SD effectively augmented the function of the Cx43 and Panx1 hemichannels, we assessed their activity by measuring the uptake of Etd. This dye enters the cytoplasm of healthy cells through plasma membrane channels with large pores, including hemichannels [63]. Etd becomes fluorescent upon intercalation with DNA and RNA base pairs, reflecting channel activity. A focal high [K^+^] puff triggered a rapid increase in Etd uptake that correlated temporally and spatially with the decrease in light transmittance observed via IOS imaging (Fig. 4A-C and Fig. 1C, D). A high [K^+^]-induced increase in Etd uptake was consistently observed throughout the recording period (Fig. 4C–E). However, it was more pronounced during the initiation phase than during the spreading phase (Fig. 4E). During the initiation phase, Gap19, ^10^panx1, or the combination of both peptides partially attenuated the high [K^+^]-induced increase in Etd uptake (Fig. 4C and E). In contrast, during the spreading phase, neither Gap19 nor ^10^panx1 affected Etd uptake, while their combination completely reduced Etd uptake (Fig. 4E). These findings indicate that high [K^+^]-induced SD increases the activity of the Cx43 and Panx1 hemichannels in the brain cortex.

**Fig. 5:**
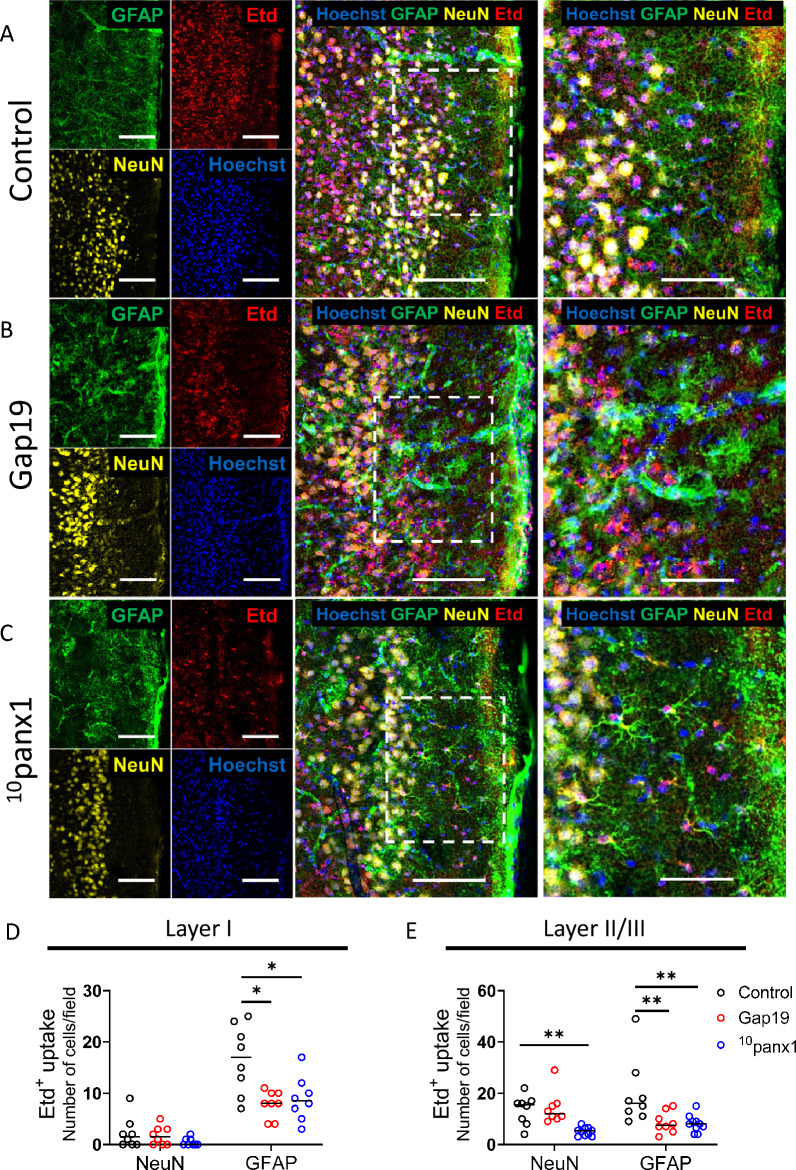
High [K^+^]-induced spreading depression activates connexin-43 and pannexin-1 hemichannels predominantly in astrocytes but also in neurons. **A**, **C** Representative confocal images showing glial fibrillary acidic protein (GFAP; green), NeuN (yellow), ethidium (Etd; red) and Hoechst (blue) staining at the site of IOS imaging after high [K^+^] puff in the cortex of control **A** or Gap19 (300 µM) **B** or ^10^panx1 (200 µM) **C** brain slices. An inset from the boxed area is shown in the right panel. Left and middle panels: Scale bar 100 µm; right panel: Scale bar 50 µm. **D**, **E** Number of Etd-positive neurons (NeuN^+^) or astrocytes (GFAP^+^) per field in brain slices stimulated with high [K^+^] puff under control conditions (white circles) or treated with Gap19 (300 µM) (red circles) or ^10^panx1** (**200 µM) (blue circles) in cortical layers I (**D**) and II/III (**E**). *P < 0.05, **P < 0.01, for the effect of Cx43 or Panx1 hemichannel inhibition compared to the respective control conditions (Mann–Whitney nonparametric test). The values are expressed as the number of cells/field. Line segments correspond to group medians. n = 8 fields

Astrocytes express functional hemichannels composed of Cx43 and Panx1 [26], whereas neurons form Panx1 hemichannels [64]. Therefore, we decided to identify the cell types showing increased hemichannel activity evoked by SDs. For that purpose, Etd uptake was evaluated during “snapshot” experiments in GFAP-positive astrocytes or Neu-N-positive neurons from cortical layers I and II/III of brain slices stimulated with focal high [K^+^] puff. Confocal microscopy revealed that high [K^+^]-induced Etd uptake was more predominant in astrocytes than in neurons in cortical layer I (Fig. 5A, D). Gap19 or ^10^panx1 strongly reduced the number of astrocytes showing Etd uptake triggered by focal high [K^+^] puff (Fig. 5A–D). In contrast, neither Gap19 nor ^10^panx1 altered the number of neurons exhibiting Etd uptake in cortical layer I (Fig. 5A–D). In cortical layers II/III, high [K^+^]-induced Etd uptake was observed in both astrocytes and neurons (Fig. 5Aand E). Notably, Gap19 strongly reduced the number of astrocytes showing Etd uptake upon focal high [K^+^] puff (Fig. 5A, B and E). Moreover, ^10^panx1 prominently mitigated the number of neurons and astrocytes that exhibited Etd uptake under the same stimulus (Fig. 5A, C and E). Overall, these results demonstrate that high [K^+^]-induced SD increases the activity of Panx1 hemichannels in astrocytes and neurons as well as the activity of Cx43 hemichannels in astrocytes in the brain cortex.

### The functional inhibition of neurons and astrocytes has opposite effects on hemichannel activation during spreading depression

**Fig. 6:**
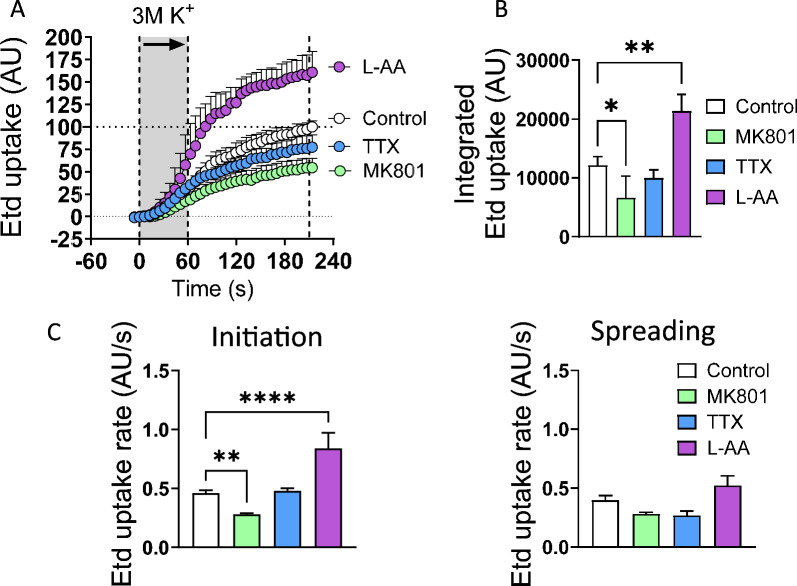
Opposite effects of the functional inhibition of astrocytes and neurons on hemichannel activity induced by high [K^+^] in the brain cortex. **A** Averaged plots of Etd uptake normalized to control condition (white circles) induced by 3 M [K^+^] puff in brain slices treated with MK801 (400 µM) (blue circles), L-AA (1 mM) (magenta circles), or TTX (1 µM) (green circles). The gray areas between the dashed lines delimit the initiation and spreading phases of the Etd uptake wave. **B** Averaged data of integrated Etd uptake induced by 3 M [K^+^] puff in brain slices under control conditions or after treatment with MK801 (400 µM), L-AA (1 mM) or TTX (1 µM). **C** Average Etd uptake rate induced by 3 M [K^+^] puff during the initiation and spreading phases in brain slices under control conditions or after treatment with MK801 (400 µM), L-AA (1 mM) or TTX (1 µM). *P < 0.05, **P < 0.01, ****P < 0.0001 for the effect of neuron or astrocyte inhibition compared to the respective control conditions during the different phases of SD (one-way ANOVA followed by Dunnet's post hoc test). The values are expressed in arbitrary units (A.U.). n = 7 mice per group

Next, we studied the differential participation of neurons and astrocytes in high [K^+^]-induced Etd uptake using MK801/TTX and L-AA, respectively. MK801 (400 µM) significantly mitigated the Etd uptake induced by focal high [K^+^] puff during initiation but not during the spreading phase (Fig. 6A–C). Conversely, L-AA prominently potentiated the high [K^+^]-induced Etd uptake during the initiation but not the spreading phase (Fig. 6A–C). Notably, although TTX tended to reduce the Etd uptake induced by focal high [K^+^] puff, this effect was not significant. These findings suggest that the inhibition of astrocytes could potentiate the activation of hemichannels evoked by high [K^+^]-induced SD, whereas the blockade of neuronal NMDA receptors has the opposite effect.

### Inhibition of Cx43 and Panx1 hemichannels mitigates the decrease in synaptic transmission evoked by spreading depression in the brain cortex and hippocampus

**Fig. 7:**
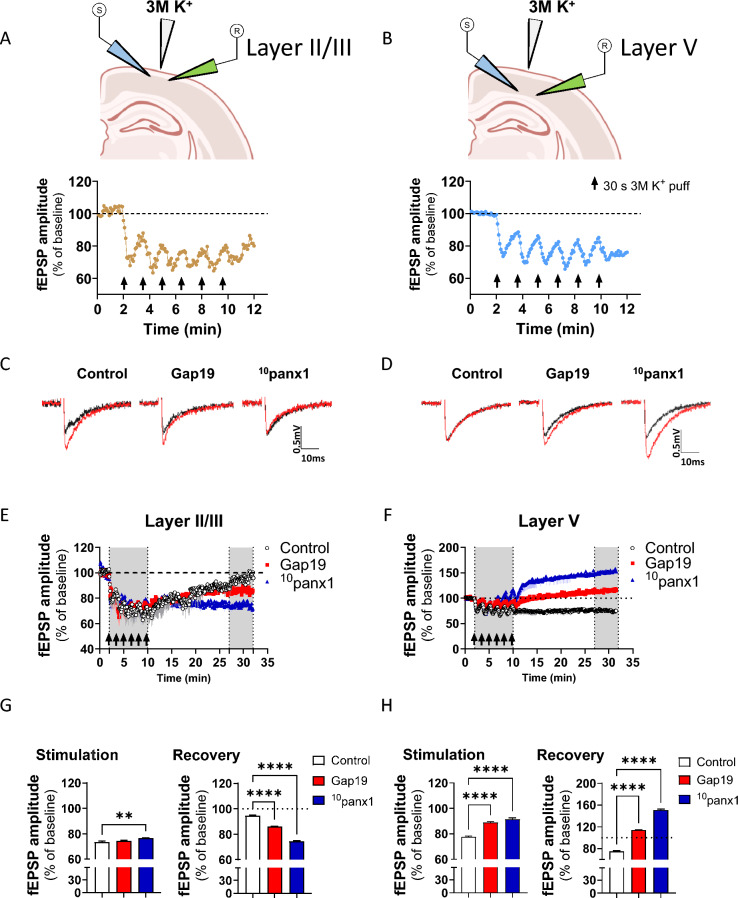
Connexin-43 and pannexin-1 hemichannels contribute to decreased synaptic transmission evoked by high [K^+^]-induced spreading depression in the brain cortex. **A**, **B** Top panels**.** Schematics showing the site of the focal train of high [K^+^] puffs as well as the position of stimulatory and recording electrodes on cortical layers II/III (**A**) and V (**B**). Bottom panels. The amplitude of fEPSPs normalized to baseline (dashed line) in brain slices during the stimulation period with a train of consecutive high [K^+^] puffs (arrows) in cortical layers II/III (**A**) and V (**B**). **C**, **D** Representative traces of fEPSPs in cortical layers II/III (**C**) and V (**D**) during high [K^+^] stimulation (black) or after 20 min of recovery (red) in brain slices under control conditions or after treatment with Gap19 (300 µM) or ^10^panx1 (200 µM). **E**, **F** Amplitude of fEPSPs normalized to baseline (dashed line) in brain slices during and after the stimulation period with a train of consecutive high [K^+^] puffs (arrows) in cortical layers II/III (**E**) and V (**F**) from brain slices under control conditions or treated with Gap19 (300 µM) or ^10^panx1 (200 µM). The gray areas delimit the stimulation period with high [K^+^] puffs and the recovery time (last 5 min of recording). **G**, **H** Averaged data of the amplitude of fEPSPs normalized to baseline (dashed line) in brain slices during and after the stimulation period with a train of consecutive high [K^+^] puffs (arrows) in cortical layers II/III (**G**) and V (**H**) from brain slices under control conditions or treated with Gap19 (300 µM) or ^10^panx1 (200 µM). **P < 0.01, ****P < 0.0001, for the effect of Cx43 or Panx1 hemichannel inhibition compared to the respective control conditions (Mann–Whitney nonparametric test). n = 6 slices per group

Because neuronal silencing is a hallmark of SD [2, 3, 42], we explored whether our ex vivo model of SD could decrease basal excitatory synaptic transmission in the brain cortex. With this in mind, we studied the amplitude of field excitatory postsynaptic potentials (fEPSPs) triggered by using our ex vivo model of SD. Pyramidal cells in cortical layers II/III establish monosynaptic connections through long horizontal collaterals with the proximal dendrites of layer II/III cells in distant columns [65]. The trend of consecutive focal high [K^+^] puffs was used to simulate an in vivo cluster of SD after acute brain injury [66]. Successive application of focal high [K^+^] puffs for eight minutes caused a rapid decrease in the amplitude of fEPSPs elicited by stimulation of these axon collaterals in layers II/III (Fig. 7A). After the last stimulation with a high [K^+^] puff, the amplitude of the potentials showed an apparent recovery (Fig. 7C, E and G). Then, to determine the contributions of the Cx43 and Panx1 hemichannels to the above phenomenon, we used Gap19 and ^10^panx1 peptides, respectively. Quantification of the fEPSPs amplitude showed that Gap19 did not alter the decrease in synaptic transmission evoked by high [K^+^] puffs during the stimulation period (Fig. 7C, E and G). However, we noted that inhibition of Panx1 hemichannels with ^10^panx1 slightly alleviated the high [K^+^]-induced reduction in fEPSPs amplitude (Fig. 7C, E and G). During the recovery period following high [K^+^] stimulation, treatment with ^10^panx1 rather than Gap19 affected the recovery rate by producing a prolonged decrease in fEPSPs amplitude (Fig. 7C, E and G).

Next, we investigated whether this synaptic modulatory effect of hemichannels during SD was a general phenomenon or specific to cortical layers. In this context, we applied a similar experimental approach in cortical layer V. Axons arising from this layer provide monosynaptic input to pyramidal cells in all layers of neighboring columns by synapsing in two dendritic fields: one in the superficial layers and the other in the middle layer [65]. Thus, we focused our recordings on middle layer V. Similar to what was found in cortical layer II/III, high [K^+^] puffs decreased the size of fEPSPs during the stimulation period in cortical layer V (Fig. 7B). Nevertheless, the amplitude of fEPSPs did not recover following 25 min of high [K^+^] puffs, maintaining an ~ 25% reduction compared to baseline (Fig. 7B, D, F and H). Interestingly, both the Gap19 and ^10^panx1 peptides drastically reduced the high [K^+^]-evoked reduction in fEPSP size during the stimulation period (Fig. 7D, F and H). More importantly, this protective effect persisted during the recovery period, even though ^10^panx1 increased the amplitude of fEPSPs compared to baseline (Fig. 7D, F and H).

**Fig. 8:**
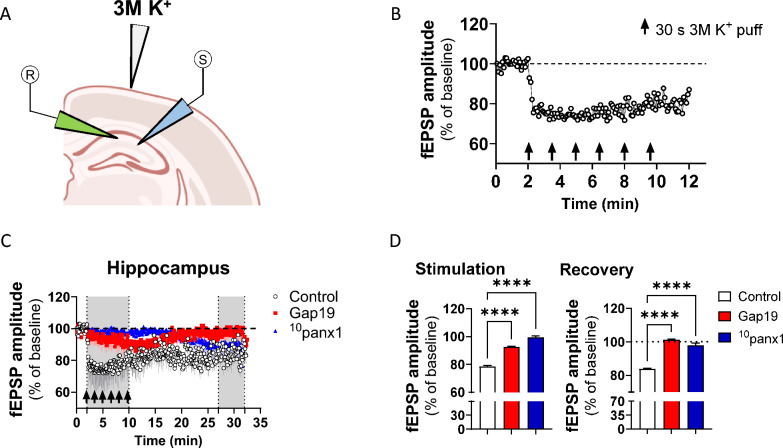
Connexin-43 and pannexin-1 hemichannels contribute to synaptic transmission decline evoked by spreading depression in the hippocampus.** A** A schematic showing the site of the focal train of high [K +] puffs and the position of stimulatory and recording electrodes on Schaffer collaterals and the stratum radiatum, respectively. **B** The amplitude of fEPSPs normalized to baseline (dashed line) in brain slices during the stimulation period with a train of consecutive high [K^+^] puffs (arrows) in the hippocampus. **C** The amplitude of fEPSPs normalized to baseline (dashed line) during and after the stimulation period with a train of consecutive high [K^+^] puffs (arrows) in the hippocampus from brain slices under control conditions or treated with Gap19 (300 µM) or ^10^panx1 (200 µM). The gray areas delimit the stimulation period with high [K^+^] puffs and the recovery time (last 5 min of recording). **D** Averaged data of the amplitude of fEPSPs normalized to baseline (dashed line) in brain slices during and after the stimulation period with a train of consecutive high [K^+^] puffs (arrows) in the hippocampus from brain slices under control conditions or treated with Gap19 (300 µM) or ^10^panx1 (200 µM). ****P < 0.0001, for the effect of Cx43 or Panx1 hemichannel inhibition compared to the respective control condition (Mann–Whitney nonparametric test). n = 6 slices per group

Finally, to analyze the remote neuroanatomical impact of cortical SD, we studied its effect on the hippocampus, a crucial area implicated in spatial memory and navigation, learning, and emotion [67]. To do this, we recorded local fEPSPs induced in the CA1 stratum radiatum by stimulating Schaffer collaterals (Fig. 8A). Notably, high [K^+^] decreased the size of fEPSPs in the CA1 stratum radiatum during the stimulation period (Fig. 8B). Moreover, similar to what was observed in cortical layer V, the amplitude of fEPSPs did not recover following 25 min of high [K^+^] puffs, maintaining an ~ 18% reduction compared to baseline (Fig. 8C–D). Surprisingly, inhibition of both the Cx43 and Panx1 hemichannels drastically reduced the high [K^+^]-evoked reduction in fEPSP amplitude in the hippocampus during the stimulation and recovery periods (Fig. 8C–D). The above results indicate that the Cx43 and Panx1 hemichannels contribute to the decrease in synaptic transmission evoked by SD in the brain cortex and hippocampus.

## Discussion

Repetitive SDs are frequently observed in association with acute brain injuries, such as subarachnoid and intracerebral hemorrhage, stroke, or TBI. These events are linked to secondary brain damage due to the substantial energy demands required to maintain ionic and neurotransmitter homeostasis, potentially resulting in poor neurological outcomes [68–70]. To date, there is no effective treatment for SD. In this study, we reported the first evidence showing that SD rapidly boosts the activity of Cx43 and Panx1 hemichannels in the brain cortex. This heightened hemichannel activation was predominantly observed in astrocytes, with neurons also demonstrating it. Importantly, the opening of Cx43 and Panx1 hemichannels was pivotal for the initiation and propagation of SDs. Furthermore, the activity of these channels critically contributed to the SD-induced decrease in synaptic transmission in the cortex and hippocampus. This suggests that hemichannels could be seen as new molecular targets for preventing the onset and spread of SD in various acute brain injuries.

Time-lapse recordings of the dynamic changes in the IOS revealed that focal high [K^+^] stimulation consistently triggered characteristic features of SD. This included a negative shift in the extracellular field potential, coupled with a wave of decreased light transmittance [10, 43, 47, 48]. The contribution of neuronal activity to this phenomenon was explored by blocking NMDA receptors or voltage-gated Na^+^ channels with MK801 and TTX, respectively. Previous studies have shown that MK801 or TTX prevents SD and blocks subsequent damage to acute brain slices [49, 71, 72]. Consistent with this finding, we observed that MK801 or TTX mitigated the spreading phase but not the initiation phase of SD. This finding implies that neuronal activity plays a crucial role in the propagation of SD, while other cellular and/or molecular pathways likely contribute to the onset of SD.

Interestingly, inhibition of astrocyte function with gliotoxin L-AA exacerbated SD propagation. Similar enhanced effects on SD have been described for another gliotoxin: fluorocitrate [73]. In fact, Largo and colleagues reported that the impairment of astrocyte function induced by fluorocitrate produces SD waves that propagate faster and last longer [73]. The latter suggests that the loss of functional astrocytes increases the susceptibility of brain tissue to SD and therefore, it may increase the risk of neuronal damage. By assessing Etd uptake, we demonstrated that high [K^+^]-induced SD predominantly increased the activity of hemichannels in astrocytes, whereas hemichannel activation was also observed in neurons. Indeed, well-established mimetic peptides known for their ability to antagonize Cx43 and Panx1 hemichannels significantly blunted SD-induced Etd uptake in astrocytes. Moreover, equivalent inhibitory effects were observed with the blockade of Panx1 hemichannels in neurons, underscoring that SD causes the activation of both hemichannels in a cell-specific manner. These data agree with previous reports documenting that neuropathological conditions increase the opening of Panx1 hemichannels in astrocytes [74] and neurons [64], as well as increase the opening of Cx43 hemichannels in astrocytes [75, 76]. Crucially, we demonstrated that blockade of Cx43 and Panx1 hemichannels prevents high [K^+^]-induced SD in a hemichannel-dependent manner. While blocking Panx1 hemichannels prevented [K^+^]-induced SD and Etd uptake during the initiation and spreading phases, inhibition of Cx43 hemichannels reduced them only during the initiation phase. Moreover, during the spreading phase of SD, blockade of Cx43 hemichannels increased the IOS above baseline, which could be associated with cell swelling [43]. These findings indicate that Cx43 hemichannels could play a tuning role in the initiation and propagation of SD.

The SD-induced activation of Panx1 neuronal hemichannels is consistent with previous evidence demonstrating in vivo activation of these channels in neurons following SD evoked by pinprick or high [K^+^] [40]. Karatas and colleagues showed that Panx1 hemichannel blockade did not prevent SD but did prevent its downstream consequences, such as inflammation and changes in meningeal artery blood flow. In addition, another study revealed that ablation of Panx1 in excitatory glutamatergic neurons does not affect SD in vivo [77]. In contrast, we found that SD activates Panx1 hemichannels not only in neurons but also in astrocytes, both of which critically contribute to the onset and propagation of SD. This discrepancy may rely on differences in experimental models (ex vivo vs. in vivo) and incubation periods of pharmacological inhibitors (45 vs. 15 min) [40].

Acute brain injuries, such as TBI, stroke, or subarachnoid hemorrhage, are affected by [K^+^]_e_ reaching > 50 mM [78–81], and are frequently reported in association with cortical SD by electrocorticography recordings [66]. Our study used a brief focal [K^+^] stimulus to trigger SD with [K^+^]_e_ levels ~ 100 × higher than those observed in pathological states. Nevertheless, this approach is commonly used as a consolidated model to obtain reproducible SDs [5, 41, 42]. This high concentration of K^+^_e_ in a small volume induces SD while ensuring that the surrounding CSF washes away K^+^_e_. This prevents its accumulation and direct effects far from the ejection point, resulting in an insignificant final change in [K^+^]_e_ (+ 0.03 mM). The mechanisms of hemichannel activation could differ depending on the type of SD inducer. For example, in hypoxia, the initial event is Na^+^/K^+^ pump failure, and the subsequent SD is terminal with no electrical recovery of the tissue [82]. On the other hand, given that Panx1 also forms GJCs [23], its ablation likely suppresses hemichannel and cytoplasmatic cell‒cell communication with potentially modulatory effects on SD propagation. In agreement with our data, findings from Chen and collaborators suggest that Panx1 hemichannels together with P2X7 receptors, participate in SD induction, subsequent cortical inflammation and trigeminovascular activation [83].

Earlier reports have shown that cortical SD leads to a temporary decrease in the amplitude of the fEPSP in the hippocampus, followed by a return to pre-SD levels [44, 84]. In agreement with this evidence, we determined that high [K^+^]-induced cortical SD reduces basal excitatory synaptic transmission in the brain cortex and hippocampus. These responses were transient in cortical layers II/III, while in cortical layer V and the hippocampus, they remained persistently reduced after 20 min of SD induction. More importantly, this long-lasting decrease in synaptic transmission was effectively mitigated by blocking the Cx43 and Panx1 hemichannels. This finding implies the involvement of these hemichannels in the synaptic depression triggered by SD in cortical layer V and the hippocampus. This finding is in line with previous studies showing that Panx1 and Cx43 hemichannels regulate basal synaptic transmission in the hippocampus and brain cortex [35, 36, 85]. Remarkably, the proposed mechanism suggests that prolonged depression of synaptic transmission after SD is triggered by the accumulation of adenosine and subsequent activation of its A1 receptor [84]. This notion coincides with the fact that Cx43 and Panx1 hemichannel activation leads to adenosine production and further activation of adenosine A1 receptors in the brain [86, 87]. Related to our study, Kawamura Jr and colleagues showed that ATP released via Panx1 hemichannels undergoes dephosphorylation to adenosine, which activates neuronal adenosine A1 receptors [87]. Surprisingly, this activation results in the hyperpolarization of the neuronal membrane potential through ATP-sensitive K^+^ channels. Taken together, this evidence supports the idea that adenosine released through hemichannels could be involved in the SD-mediated inhibition of synaptic transmission in our system. These results are clinically relevant because acute brain injuries can lead to persistent cognitive deficits and poor long-term neurological outcomes, possibly in part due to the "silent" presence of SDs [66, 68–70]. What is the mechanism underlying SD, and how do hemichannels contribute to it? Computational modeling suggests that a rapid increase in [K^+^]_e_ beyond a certain threshold triggers a positive feedback loop, initiating a self-sustaining wave of depolarization in SD [88]. Certainly, there is an inverse relationship between the [K^+^]_e_ threshold and the compromised area [89]. Thus, an initial "critical mass" is required to trigger SD. Both Cx43 and Panx1 hemichannels are activated in vitro or in vivo in response to increases in [K^+^]_e_ [57, 90–92]. The mechanism through which high [K^+^]_e_ activates Cx43 hemichannels remains unknown. However, some evidence has shed light on the activation of Panx1 hemichannels in a similar context. Under voltage-clamp conditions, high [K^+^]-induced stimulation of Panx1 hemichannel currents still occurs, suggesting that Panx1 activation is not solely due to membrane depolarization caused by increased [K^+^]_e_ [92]. Indeed, a direct association of the K^+^ ion with the first extracellular loop of Panx1 has been proposed [90]. Further studies are needed to elucidate how a high [K^+^] increases the activation of hemichannels during SD. In addition, we speculate that the contribution of hemichannels to the initiation and propagation of SD could be related to ionic and water homeostasis imbalances. Indeed, SD is linked to nonselective cation influx (e.g., Na^+^, K^+^, and Ca^2+^) [4], and hemichannels participate in this process under different pathological conditions [62, 93–95]. Electrophysiological recordings have shown that cationic inward currents during SDs are crucial for this phenomenon [96, 97]. The latter has been supported by computational modeling of SD [88]. The influx of Ca^2+^ is particularly noteworthy because Ca^2+^ serves as a natural second messenger to cells and is linked to the vasoconstriction observed after SD [98]. Specifically, Cx43 hemichannels are permeable to Ca^2+^ [99–101], and Panx1 hemichannels can indirectly increase intracellular Ca^2+^ through the release of ATP and further P2 receptor activation [102, 103]. In fact, under pathological conditions, the exacerbated opening of hemichannels leads to nonselective cation currents that induce cell swelling and further cell death [62, 94, 104]. Overall, these data suggest that hemichannels likely contribute to SD by altering the ionic and water balance and releasing high amounts of paracrine molecules that could affect the excitability of neurons (e.g., ATP and its subproducts, glutamate or D-serine).

## Conclusion

The current study revealed a novel mechanism contributing to the pathogenesis and progression of neurological disorders in which SD plays a crucial role in its etiology. We demonstrated that the Cx43 and Panx1 hemichannels participate in SD induced by a pulse of high [K^+^]_e_ in an acute brain slice model. We propose both hemichannels as novel molecular targets to prevent the initiation and propagation of SD in several acute brain injuries.

### Supplementary Information

Supplementary Material 1. Figure S1. Nonspecific connexin-43 and pannexin-1 hemichannel blockers prevent the spreading depression evoked by high [K^+^] in the brain cortex.

## Data Availability

All data generated or analyzed during this study are included in this published article.

## Abbreviations

aCSF: Artificial cerebrospinal fluid
AMPA: Alpha-amino-3-hydroxy-5-methyl-4-isoxazolepropionic acid
BBB: Blood–brain barrier
CNS: Central nervous system
Cx43: Connexin-43
Etd: Ethidium
fEPSPs: Field excitatory postsynaptic potentials
GFAP: Glial fibrillary acidic protein
GJCs: Gap junctional channels
IOS: Intrinsic optical signal
L-AA: L-2 aminoadiptic acid
NMDA: *N*-Methyl-D-aspartate
Panx1: Pannexin-1
PTX: Picrotoxin
ROI: Region of interest
SD: Spreading depression
TBI: Traumatic brain injury
TTX: Tetrodotoxin
