# Supplementary material for: Inhibition of astroglial hemichannels prevents synaptic transmission decline during spreading depression
Source: Biol Res. 2024 Jun 12;57:39. doi: 10.1186/s40659-024-00519-9 (PMC11167948; doi:10.1186/s40659-024-00519-9)
Supplement: Supplementary file 1 — Supplementary Material 1. Figure S1. Nonspecific connexin-43 and pannexin-1 hemichannel blockers prevent the spreading depression evoked by high [K+] in the brain cortex. [file 40659_2024_519_MOESM1_ESM.pdf]

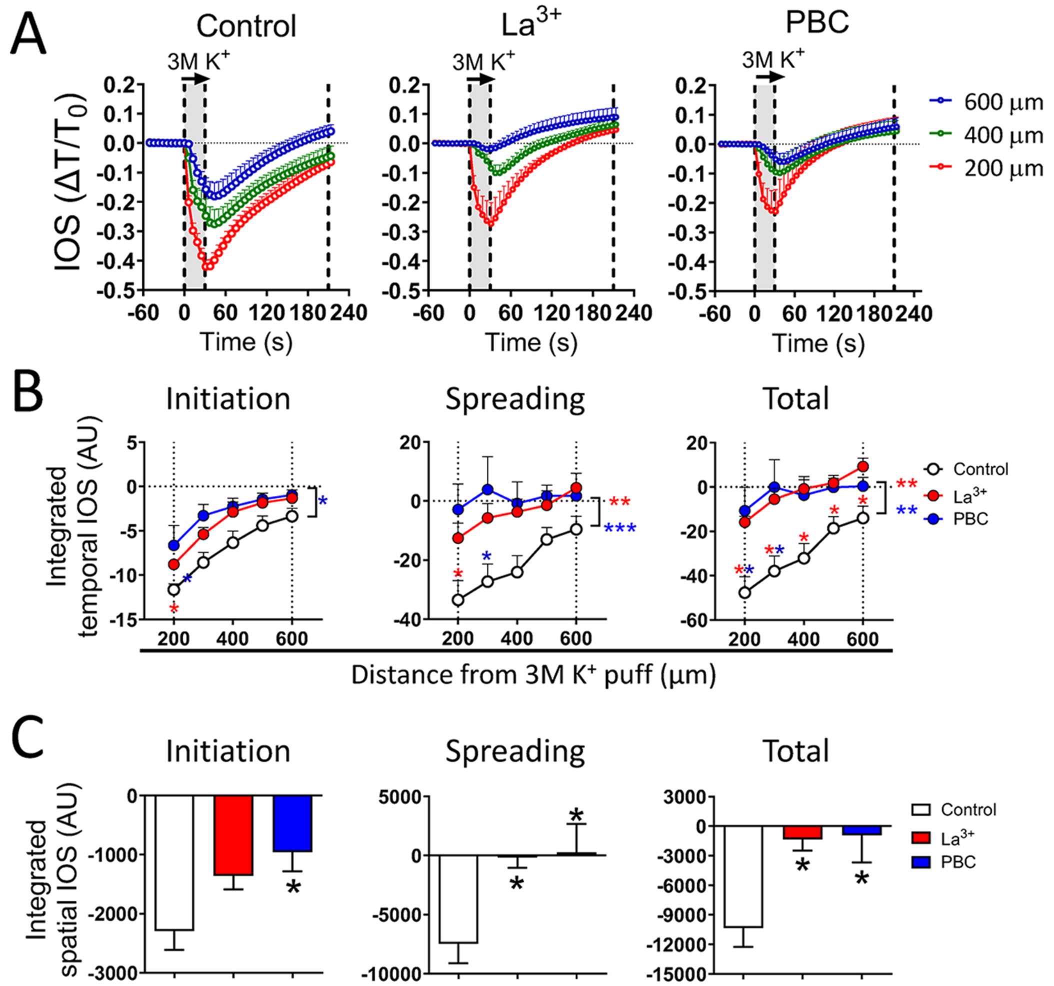

Supplementary Figure 1. Nonspecific connexin-43 and pannexin-1 hemichannels blockers prevent the spreading depression evoked by high [K$^+$] in the brain cortex. A, Representative plots of relative changes in IOS over time induced by 3 M [K$^+$] puff at different distances from the site of stimulation in brain slices under control conditions or treated with lanthanum ion (La^{3+}, 200 µM) or probenecid (PBC, 500 µM). The gray areas between the dashed lines delimit the initiation and spreading phases of IOS wave. **B,** Averaged data of integrated temporal IOS (from plots in A) induced by 3 M [K$^+$] puff during the initiation, spreading or both phases of SD wave at different distances from the site of stimulation in brain slices under control conditions or treated with lanthanum ion (La^{3+}, 200 µM) or probenecid (PBC, 500 µM). *$P<0.05$, **$P<0.01$, ***$P<0.001$, for the effect of nonspecific Cx43 or Panx1 hemichannel inhibition compared to respective control condition in each ROI during the different phases of IOS wave (Mann–Whitney nonparametric test). **C,** Averaged data of integrated spatial IOS (from plots in B) induced by 3 M [K$^+$] puff during the initiation, spreading, or both phases of SD wave at different distances from the site of stimulation in brain slices under control conditions or treated with lanthanum ion (La^{3+}, 200 µM) or probenecid (PBC, 500 µM). *$P<0.05$ for the effect of nonspecific Cx43 or Panx1 hemichannel inhibition compared to respective control condition during the different phases of IOS wave (Mann–Whitney nonparametric test). Values are expressed in arbitrary units (A.U.). Control, n= 6; La^{3+}, n= 4; PBC, n= 4, mice per group.
